# Prospects of Vitamin C as an Additive in Plasma of Stored Blood

**DOI:** 10.1155/2015/961049

**Published:** 2015-08-09

**Authors:** R. Vani, R. Soumya, H. Carl, V. A. Chandni, K. Neha, B. Pankhuri, S. Trishna, D. P. Vatsal

**Affiliations:** Center for Post Graduate Studies, Jain University, No. 18/3, 9th Main, 3rd Block, Jayanagar, Bangalore 560011, India

## Abstract

There is a dire necessity to improve blood storage and prolong shelf-life of blood. Very few studies have focused on oxidative stress (OS) in blood and its influence on plasma with storage. This study attempts to (i) elucidate the continuous changes occurring in plasma during storage through oxidant levels and antioxidant status and (ii) evaluate the influence of vitamin C (VC) as an additive during blood storage. Blood was drawn from male *Wistar* rats and stored for 25 days at 4°C. Blood samples were divided into control and experimental groups. Plasma was isolated every 5 days and the OS markers, antioxidant enzymes, lipid peroxidation, and protein oxidation products, were studied. Catalase activity increased in all groups with storage. Lipid peroxidation decreased in VC (10) but was maintained in VC (30) and VC (60). Although there were variations in all groups, carbonyls were maintained towards the end of storage. Advanced oxidation protein products (AOPP) increased in VC (30) and were maintained in VC (10) and VC (60). Sulfhydryls were maintained in all groups. Vitamin C could not sufficiently attenuate OS and hence, this opens the possibilities for further studies on vitamin C in combination with other antioxidants, in storage solutions.

## 1. Introduction

Blood transfusion is an irreplaceable, lifesaving, and overall safe treatment. Continued developments in storage techniques have resulted in improved storage and blood quality. Whole blood is stored in CPDA (citrate phosphate dextrose and adenine) or ACD (acid, citrate, and dextrose) solution up to a period of 35 days at 4°C [1]. The storage of blood in* ex vivo* conditions causes biochemical and biomechanical changes (storage lesion), which in turn affect optimal functioning and survival [2–6]. Transfusions of these altered products are associated with increased morbidity and mortality [7]. However, the alterations that occur during storage appear to be partially reversible by use of improved storage conditions and additive solutions [8]. Hence, better storage will require a system that will provide critical nutrients, improve storage milieu, and reduce the stress of storage.

One of the reasons for the formation of the storage lesion is oxidative stress (OS). This was evident in our earlier study on erythrocytes of stored blood [9]. During storage, erythrocytes undergo structural and functional changes that reduce the viability of cells. These changes include variations in the levels of the endogenous and exogenous antioxidant system and oxidative modifications of (i) proteins (protein carbonyls, advanced oxidation protein products, and protein sulfhydryls) and (ii) lipids (thiobarbituric acid reactive substances) in the erythrocyte membrane which destabilize its structure [10, 11]. The lifespan of rat erythrocytes in circulation (60 days) is lower when compared to humans (120 days). Thus, rat erythrocytes undergo deterioration more rapidly than human erythrocytes. The storage lesion in rat erythrocytes stored for a week is similar to that in human erythrocytes stored for 4 weeks [12]. Hence, studying rat erythrocytes would provide an insight into the OS situation during storage.

There are many efficient antioxidants which can reduce the OS induced by storage [13–15]. For example, antioxidant effects of vitamin C (ascorbic acid) have been demonstrated in many experiments* in vitro* [16]. It is regarded as the most important water-soluble antioxidant in plasma [17], has been shown to neutralize reactive oxygen species (ROS), and reduces OS [18, 19]. In addition to scavenging ROS and reactive nitrogen species, vitamin C can regenerate other small molecule antioxidants, such as *α*-tocopherol, glutathione (GSH), urate, and *β*-carotene, from their respective radical species [20]. Studies have reported the various changes that occur in different storage solutions, the effect of curcumin on plasma [15] and the effect of vitamin C on storage in erythrocytes [21–25]. However, the utilization of plasma as a mode of assessing the changes in blood, during storage with ascorbic acid as an additive, has not been explored. Plasma is a natural environment for blood morphological components. Thus, any change occurring in the blood cells is reflected in the plasma and thereby gives an insight into the condition of stored blood.

Therefore, we aimed to study two aspects (i) the continuous changes occurring during storage and (ii) the influence of vitamin C as an additive in stored blood. The changes occurring in plasma isolated from stored blood were analyzed at regular intervals during a period of 25 days.

In this regard the following objectives were put forth:

- to analyze the antioxidant status of plasma through antioxidant enzymes: superoxide dismutase (SOD) and catalase (CAT),
- to evaluate the oxidant levels through lipid peroxidation (thiobarbituric acid reactive substances (TBARS)) and protein oxidation (protein carbonyls (PrC), advanced oxidation protein products (AOPP), and protein sulfhydryls (P-SH)),
- to determine the effects of ascorbic acid as an additive in storage solution.

## 2. Materials and Methods

### 2.1. Animals

Male* Wistar* rats were maintained till 4 months of age, in accordance with the ethical committee regulations. Five animals were maintained for each group. Animals were lightly anaesthetized with ether and restrained in dorsal recumbency as described earlier [26]. In brief, the syringe needle was inserted just below the xiphoid cartilage and slightly to the left of midline. 4-5 mL of blood was carefully aspirated from the heart into collecting tubes with CPDA-1 (citrate, phosphate, dextrose, and adenine).

### 2.2. Chemicals

Epinephrine, thiobarbituric acid, and bovine serum albumin (BSA) were purchased from Sigma-Aldrich Chemicals (St. Louis, MO, USA). All other chemicals used were of reagent grade and organic solvents were of spectral grade.

### 2.3. Experimental Design

Blood was drawn from male* Wistar* rats (4 months old) and stored over a period of 25 days at 4°C in CPDA-1. Blood samples were divided into two groups: controls and experimentals. Ascorbic acid of varying concentrations was added to the experimental group: 10 mM, 30 mM, and 60 mM, that is, VC (10), VC (30), and VC (60) groups. Each group consisted of samples from 5 animals. Whole blood (1 mL) was aliquoted from the stored blood every fifth day and the plasma was isolated to analyze the previously mentioned parameters.

### 2.4. Plasma Separation

Plasma was isolated in Eppendorf tubes by centrifuging in a fixed angle rotor for 20 min at 2000 ×g. The plasma was removed and suspended in an equal volume of isotonic phosphate buffer, pH 7.4 [27].

### 2.5. Superoxide Dismutase (SOD, EC 1.15.1.1)

SOD was measured by the method of Misra and Fridovich [28]. Plasma was added to carbonate buffer (0.05 M). Epinephrine was added to the mixture and measured spectrophotometrically at 480 nm. SOD activity was expressed as the amount of enzyme that inhibits oxidation of epinephrine by 50%.

### 2.6. Catalase (CAT, EC 1.11.1.6)

CAT was determined by the method of Aebi [29]. Briefly, plasma with absolute alcohol was incubated at 0°C. An aliquot was taken up with 6.6 mM H_2_O_2_ and decrease in absorbance was measured at 240 nm. An extinction coefficient of 43.6 M cm^−1^ was used to determine enzyme activity.

### 2.7. Thiobarbituric Acid Reactive Substances (TBARS)

TBARS was determined by the method of Bar-Or et al. [30]. Plasma with 0.9% NaCl was incubated at 37°C for 20 min. 0.8 M HCl containing 12.5% TCA and 1% TBA was added and kept in boiling water bath for 20 min and cooled at 4°C. Centrifugation was carried out at 1500 ×g and absorbance was measured at 532 nm.

### 2.8. Protein Carbonyls (PrC)

PrC was measured as an index of protein oxidation as described by Uchida and Stadtman [31]. Protein carbonyl content was measured by forming labeled protein hydrazones derivative, using 2,4-dinitrophenyl hydrazine (DNPH), which were then quantified spectrophotometrically. Briefly after precipitation of protein with equal volume of 1% trichloroacetic acid (TCA), the pellet was resuspended in 10 mM DNPH. Samples were kept in dark for 1 h. An equal volume of 20% TCA was added and left in ice for 10 min and centrifuged at 1900 ×g and pellet was washed with ethanol-ethylacetate mixture (1 : 1) to remove the free DNPH and lipid contaminants. Final pellet was dissolved in 8 M guanidine HCl in 133 mM tris and absorbance was measured at 370 nm. The results were expressed as *μ*mol of 2,4-DNPH incorporated/mg protein based on a molar extinction coefficient of 2.1 × 10^4^ M cm^−1^ for aliphatic hydrazones.

### 2.9. Advanced Oxidation Protein Products (AOPP)

Spectrophotometric determination of AOPP levels was assayed as an index of dityrosine containing cross-linked protein products by Witko's method [32]. Plasma was diluted in phosphate buffered saline and 1.16 mol/L potassium iodide was added, followed by the addition of acetic acid. The absorbance of reaction mixture was immediately read at 340 nm. AOPP was calculated by using the extinction coefficient of 26 mM^−1^ cm^−1^.

### 2.10. Protein Sulfhydryls (P-SH)

The concentration of P-SH was measured as described by Habeeb [33]. In brief, 0.08 mol/L sodium phosphate buffer containing 0.5 mg/mL of Na_2_-EDTA and 2% SDS were added to each assay tube. 0.1 mL of 5,5′-dithiobis-(2-nitrobenzoic acid) (DTNB) was added and the solution was vortexed. Color was allowed to develop at room temperature and absorbance was measured at 412 nm. P-SH was calculated from the net absorbance and molar absorptivity, 13,600 mol L^−1^ cm^−1^.

### 2.11. Protein Determination

Protein was determined in the plasma by the method of Lowry et al. [34], using bovine serum albumin as the standard.

### 2.12. Statistical Analyses

Results are represented as mean ± SE. Values between the groups were analyzed by two-way ANOVA and were considered significant at *P* < 0.05. Bonferroni Post test was performed for antioxidant enzymes, SOD and CAT, lipid peroxidation product, TBARS, and protein oxidation products, PrC, AOPP, and P-SH concentrations using Graph Pad Prism 6 software.

## 3. Results

### 3.1. Superoxide Dismutase

SOD variation was insignificant during the storage period though increments of 100%, 300%, and 200% were observed in controls on days 10, 15, and 20, respectively against day 0.

Significant differences were observed in vitamin C groups. On day 15, SOD decreased by 75% in VC (30), whereas it increased by 200% on day 25 with respect to control. In addition, increments of 100% and 300% were also observed in VC (60) against VC (10) and VC (30), respectively.

**Figure 1:**
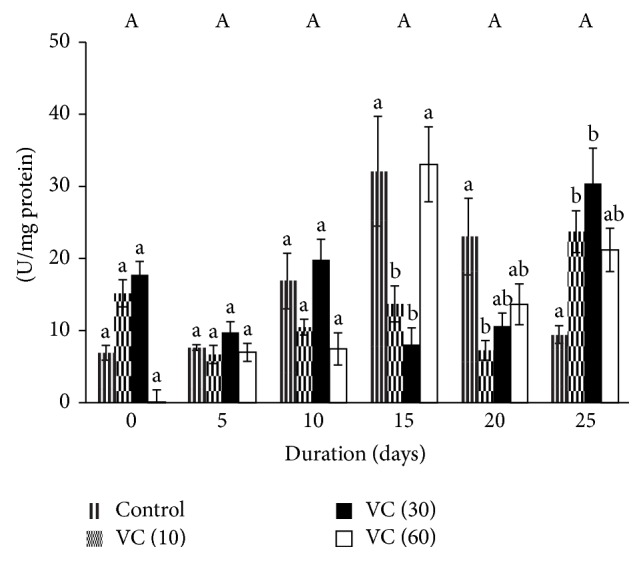
Superoxide dismutase activity in plasma isolated from stored blood. Values are mean ± SE of five animals/group. VC (10): vitamin C (10 mM); VC (30): vitamin C (30 mM); and VC (60): vitamin C (60 mM). Two-way ANOVA was performed between the groups and subgroups. Changes between the groups are insignificant. Changes within the groups are represented in lower case. Those not sharing the same letters are significantly different.

VC (10) and VC (30) showed variations in SOD activity but increased towards the end of storage. SOD in VC (60) showed an increase with storage (Figure 1).

### 3.2. Catalase

Catalase varied significantly with the storage. The activity increased in controls by 13-, 41-, 42-, and 18-fold on days 5, 15, 20, and 25, respectively, when compared to day 0. Similarly, increments of 6-fold were seen on days 5, 15, 20, and 25 in VC (10) and 12-fold (day 15) and 23-fold (days 20 and 25) in VC (30). CAT increased by 20-fold on days 20 and 25 in VC (60) when compared to day 0.

Variations in CAT between different concentrations were insignificant.

**Figure 2:**
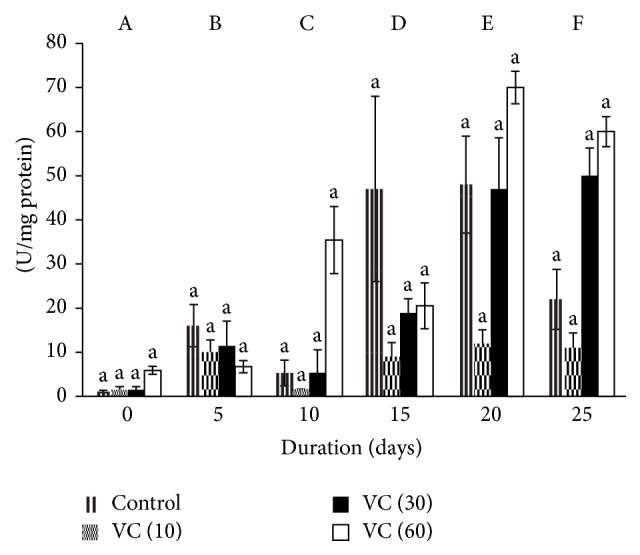
Catalase activity in stored plasma. Values are mean ± SE of five animals/group. VC (10): vitamin C (10 mM), VC (30): vitamin C (30 mM), and VC (60): vitamin C (60 mM). Two-way ANOVA was performed between the groups and subgroups. A–F values between the groups are significantly different at *P* < 0.05. Changes within the groups are represented in lower case. Those not sharing the same letters are significantly different.

Catalase activity increased in all groups with storage (Figure 2).

### 3.3. Thiobarbituric Acid Reactive Substances (TBARS)

Significant changes were observed in TBARS during storage. In controls, TBARS decreased by 80% and 40% on days 5 and 20, respectively, whereas they increased by 160% and 60% on days 10 and 15, respectively, when compared to day 0. TBARS also reduced on days 10, 20, and 25 by 40%, 90%, and 80%, respectively, and increased by 100% on day 5 when compared to day 0 in VC (10) samples. There were increments of 300% (day 5) and 100% (day 25) in VC (30) and decrements of 42%, 85%, and 65% on days 5, 10, and 25, respectively, in VC (60) against day 0.

TBARS elevated by 3-fold on day 0 in VC (60) with VC (30). On day 5, TBARS increased by 12-fold in VC (30) whereas on day 10, it decreased by 77% in VC (10), VC (30), and VC (60) when compared to control.

**Figure 3:**
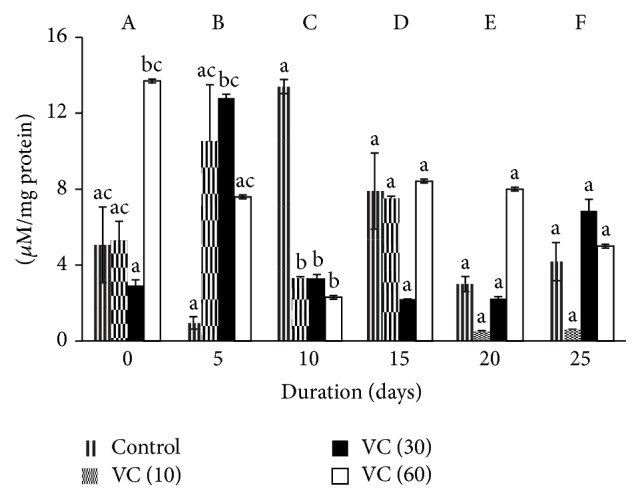
Thiobarbituric acid reactive substances in plasma isolated from stored blood. Values are mean ± SE of five animals/group. VC (10): vitamin C (10 mM), VC (30): vitamin C (30 mM), and VC (60): vitamin C (60 mM). Two-way ANOVA was performed between the groups and subgroups. A–F values between the groups are significantly different at *P* < 0.05. Changes within the groups are represented in lower case. Those not sharing the same letters are significantly different.

TBARS decreased in VC (10) but was maintained in VC (30) and VC (60) with storage (Figure 3).

### 3.4. Protein Carbonyls (PrC)

Carbonyls of controls increased significantly by 1-, 2-, 12-, 8-, and 2-fold, respectively, from days 5 to 25 with respect to day 0. In VC (10), decrements of 43%, 89%, 42%, and 91% were observed on days 5, 15, 20, and 25, respectively, while an increment of 72% was observed on day 10 against day 0. A similar trend was noticed in VC (30) as PrC reduced by 85%, 77%, 48%, and 95% on days 5, 10, 15, and 25. But, in VC (60), PrC showed increments of 176%, 71% and 62%, and 80% on days 10, 15, and 20 and 25, respectively, with respect to control.

PrC increased by 23-fold on day 0 in VC (30) with respect to control, while it decreased by 1-fold in VC (60) against VC (30) on day 0.

**Figure 4:**
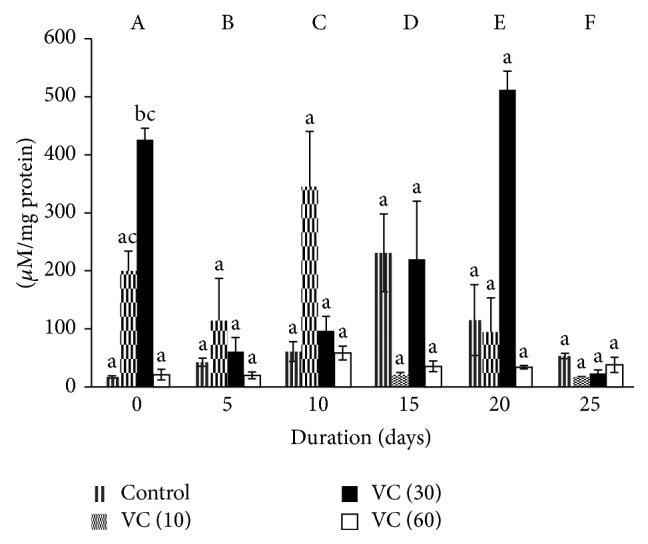
Protein carbonyls in plasma isolated from stored blood. Values are mean ± SE of five animals/group. VC (10): vitamin C (10 mM), VC (30): vitamin C (30 mM). and VC (60): vitamin C (60 mM). Two-way ANOVA was performed between the groups and subgroups. A–F values with different superscripts between groups are significantly different at *P* < 0.05. Changes within the groups are represented in lower case. Those not sharing the same letters are significantly different.

Although there were variations in the levels of PrC in all groups, it was maintained towards the end of storage (Figure 4).

### 3.5. Advanced Oxidation Protein Products (AOPP)

AOPP increased by 300% on days 15 and 25 and by 400% on day 20 in controls. AOPP also increased by 100% (days 5, 10, and 15), 200% (day 20), and 300% (day 25) in VC (10). A similar trend was observed in VC (30) as AOPP increased by 100% on days 15 and 25, 70% on day 5, and 200% on day 20. AOPP elevated by 100% on day 10 and 200% on day 15 in VC (60) with respect to control.

AOPP reduced by 69% and 72% on days 15 and 25, respectively, in VC (10) against control. AOPP elevated by 100% and 200% on days 15 and 20, respectively, in VC (30) in comparison with VC (10). Increments of 69% and 74% were observed on days 20 and 25 when VC (30) was compared with VC (60). Decrements of 72% and 74% on days 20 and 25 were observed in VC (60) against controls.

**Figure 5:**
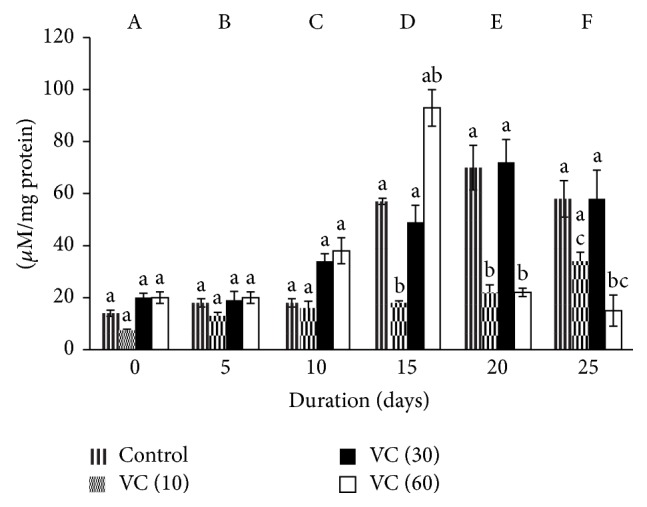
Advanced oxidation protein products in plasma isolated from stored blood. Values are mean ± SE of five animals/group. VC (10): vitamin C (10 mM), VC (30): vitamin C (30 mM), and VC (60): vitamin C (60 mM). Two-way ANOVA was performed between the groups and subgroups. A–F values between the groups are significantly different at *P* < 0.05. Changes within the groups are represented in lower case. Those not sharing the same letters are significantly different.

AOPP increased in VC (30) and was maintained in VC (10) and VC (60) (Figure 5).

### 3.6. Protein Sulfhydryls (P-SH)

Sulfhydryls varied significantly during storage. P-SH increased in controls by 3-, 2-, 8-, 10-, and 9-fold on days 5, 10, 15, 20, and 25, respectively, with day 0. P-SH also elevated by approximately 4-fold on days 5, 10, and 20, and 45-fold on day 15, respectively, in VC (30), when compared to day 0. An increment of 6-fold on day 15 and 1-fold on days 20 and 25 and a decrement of 54% were observed in VC (60) on day 10 against day 0.

On day 15, increases of 1-, 7-, and 3-fold were observed in VC (60) when compared with control, VC (10), and VC (30), respectively. On day 25, decrements of 1-fold were observed in VC (10) and VC (30) against controls.

**Figure 6:**
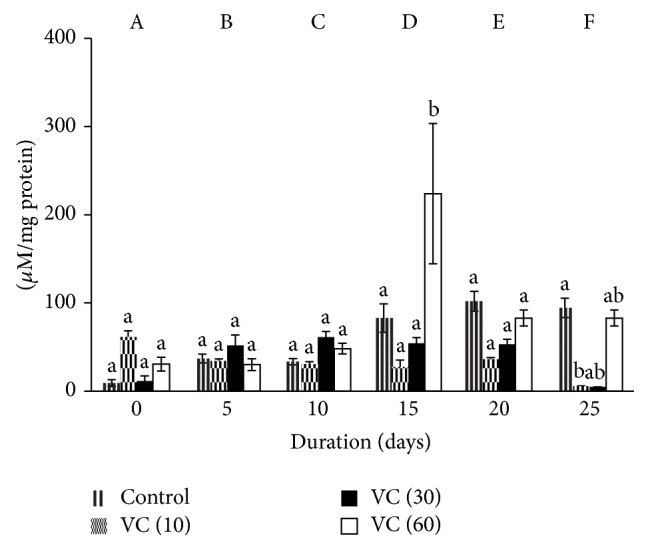
Protein sulfhydryls in plasma isolated from stored blood. Values are mean ± SE of five animals/group. VC (10): vitamin C (10 mM); VC (30): vitamin C (30 mM), and VC (60): vitamin C (60 mM). Two-way ANOVA was performed between the groups and subgroups. A–F values between the groups are significantly different at *P* < 0.05. Changes within the groups are represented in lower case. Those not sharing the same letters are significantly different.

Sulfhydryls were maintained in all groups throughout storage (Figure 6).

## 4. Discussion

The effects of vitamin C as an additive in blood during storage were evaluated through plasma. Although SOD levels were insignificant during storage, VC (10) and VC (30) decreased SOD levels on the days when ROS was found to be higher [35]. Catalase activity increased in all groups. Levels of TBARS, PrC, AOPP, and P-SH were maintained in all groups.

Blood plasma is considered well equipped with both chain-breaking and preventive antioxidants to cope with OS and prevent peroxidative damage to circulating lipids. The antioxidants do not exert their functions by merely scavenging radicals but also by inducing/activating enzymes counteracting OS or by modulating redox-sensitive metabolic pathways.

Superoxide dismutases are enzymes that convert superoxide radical to oxygen and hydrogen peroxide. These enzymes carry out catalysis via general mechanism that involves the sequential reduction and oxidation of the metals like copper, iron, manganese, and nickel, at the active site [36]. The upregulation of SOD activity indicates an increase in free radicals during storage of blood, but a decrement in vitamin C samples (10 mM and 30 mM) may be due to the antioxidant property of ascorbic acid. Ascorbic acid is a soluble, strongly reducing agent that can react directly with free radicals, thereby resulting in decreased SOD in VC (10) and VC (30). The dismutation of superoxide radical yields hydrogen peroxide. This reaction occurs spontaneously or is catalyzed by superoxide dismutases. The high reactivity of H_2_O_2_ *in vivo* is largely explained by the Fenton reaction, where H_2_O_2_ reacts with partially reduced metal ions such as Fe^2+^ or Cu^+^, to form the hydroxyl radical. This reaction can be sustained* in vitro* by the presence of mild reducing agent such as ascorbic acid that recycles the oxidized metal ions [37]. At higher concentrations, the ratio of ascorbate monoions is higher than that of the ascorbyl radical, thereby driving the Fenton reaction [38]. This may be the reason for increased SOD activity in VC (60).

Catalase rapidly catalyzes the decomposition of hydrogen peroxide to less reactive gaseous oxygen and water molecules. CAT exhibits a high *K*_*m*_ for H_2_O_2_ and can act upon H_2_O_2_ produced before it diffuses to other parts of the cell [39]. CAT may be uniquely suited to regulate the homeostasis of H_2_O_2_ in the cell. CAT activity was upregulated on all the days in the plasma of controls and vitamin C samples. This indicates that there is the formation of hydrogen peroxide, as CAT acts predominantly when H_2_O_2_ concentrations are enormously high. This also suggests that the endogenous antioxidants like glutathione, along with the vitamin C, could not attenuate the oxidative stress efficiently.

TBARS increased in the earlier stage of storage period but later decreased in controls. The earlier increase may be correlated to the latent phase of antioxidant activation and the decrease may be justified by the amelioration of the endogenous antioxidant system in the plasma. Vitamin C (ascorbic acid) is an important antioxidant in human plasma, where it acts as a scavenger of free radicals and protects against lipid peroxidation. Ascorbate plays a pivotal role in protecting plasma lipids from peroxidative damage initiated by aqueous peroxyl radicals [40, 41]. This was evident in our study as TBARS initially decreased in all vitamin C groups but later normalized to that of controls. This return to normalcy could be due to the unavailability of reduced ascorbate [42].

The quantification of oxidative damage to proteins has been studied almost exclusively by assessing the total carbonyl content. The oxidants responsible for carbonyl formation within the proteins* in vivo* are believed to be radicals, such as hydroxyl radicals. Indeed, hydroxyl radicals can be generated by metal-catalyzed oxidation systems and these systems convert several amino acid residues to carbonyl derivatives. It is known that an increase in carbonyl content reflects the oxidation of lysine, arginine, and proline residues of the proteins [43, 44].

Oxidation of proteins can lead to a whole variety of amino acid modifications. Action of chloraminated oxidants, mainly hypochlorous acid and chloramines, produced by myeloperoxidase, forms dityrosine containing cross-linked protein products known as AOPP and is also considered as one of the biomarkers to estimate the degree of oxidative modifications of proteins [36, 45].

Our results on carbonyls in controls proved that during storage period, there was production of ROS leading to oxidant damage of proteins. Ascorbyl-free radical reductase increases the ascorbic acid recycling in human plasma and is reported as a compensatory/protective mechanism that operates to maintain the ascorbic acid level in plasma and thereby minimize OS [46]. Vitamin C maintained carbonyls and AOPP as evident in our results.

Plasma is endowed with an array of antioxidant defense mechanisms. One of the important plasma antioxidants appears to be ascorbate. Protein sulfhydryl groups have also been suggested to contribute significantly to the antioxidant capacity of plasma. In particular, oxidative modification of sulfhydryl groups in proteins can be a two-faceted process: it could lead to impairment of protein function or, depending on the redox state of cysteine residues, may activate specific pathways involved in regulating key cell functions [47].

Oxidation of sulfhydryls of the membrane protein to disulfides causes reversible changes. This may be due to the disulfide exchange reactions carried out by a class of thioltransferases that catalyze reactions between glutathione and thioredoxin to regenerate the protein sulfhydryls [48]. These may be the possible reasons for variations in sulfhydryls during the storage.

## 5. Conclusion

Plasma has an efficient antioxidant system and can minimize the levels of oxidants during storage of 25 days. Vitamin C at the concentrations of 10, 30, and 60 mM also enhanced the antioxidant defenses but could not protect susceptible protein groups. Our study gives an insight into the interactions of different oxidants and antioxidants (both endogenous and exogenous). Vitamin C alone could not sufficiently attenuate OS and hence this opens the possibilities for further studies on vitamin C in combination with other antioxidants, in storage solutions.

## References

[1] Hess J. R. (2010). Conventional blood banking and blood component storage regulation: opportunities for improvement. *Blood Transfusion*.

[2] Tinmouth A., Fergusson D., Yee I. C., Hébert P. C. (2006). Clinical consequences of red cell storage in the critically ill. *Transfusion*.

[3] Yoshida T., Shevkoplyas S. S. (2010). Anaerobic storage of red blood cells. *Blood Transfusion*.

[4] Yoshida T., AuBuchon J. P., Dumont L. J. (2008). The effects of additive solution pH and metabolic rejuvenation on anaerobic storage of red cells. *Transfusion*.

[5] D'Amici G. M., Mirasole C., D'Alessandro A., Yoshida T., Dumont L. J., Zolla L. (2012). Red blood cell storage in SAGM and AS3: a comparison through the membrane two-dimensional electrophoresis proteome. *Blood Transfusion*.

[6] Antonelou M. H., Kriebardis A. G., Stamoulis K. E., Economou-Petersen E., Margaritis L. H., Papassideri I. S. (2010). Red blood cell aging markers during storage in citrate-phosphate-dextrose- saline-adenine-glucose-mannitol. *Transfusion*.

[7] Kelher M. R., Masuno T., Moore E. E. (2009). Plasma from stored packed red blood cells and MHC class I antibodies causes acute lung injury in a 2-event *in vivo* rat model. *Blood*.

[8] Hillier C., Silberstein L., Ness P. (2007). *Blood Banking and Transfusion Medicine*.

[9] Rajashekharaiah V., Koshy A. A., Koushik A. K. (2012). The efficacy of erythrocytes isolated from blood stored under blood bank conditions. *Transfusion and Apheresis Science*.

[10] Kücükakin B., Kocak V., Lykkesfeldt J. (2011). Storage-induced increase in biomarkers of oxidative stress and inflammation in red blood cell components. *Scandinavian Journal of Clinical and Laboratory Investigation*.

[11] Sachan D. S., Hongu N., Johnsen M. (2005). Decreasing oxidative stress with choline and carnitine in women. *Journal of the American College of Nutrition*.

[12] D'almeida M. S., Jagger J., Duggan M., White M., Ellis C., Chin-Yee I. H. (2000). A comparison of biochemical and functional alterations of rat and human erythrocytes stored in CPDA-1 for 29 days:implications for animal models of transfusion. *Transfusion Medicine*.

[13] Knight J. A., Searles D. A. (1994). The effects of various antioxidants on lipid peroxidation in stored whole blood. *Annals of Clinical and Laboratory Science*.

[14] Arduini A., Holme S., Sweeney J. D., Dottori S., Sciarroni A. F., Calvani M. (1997). Addition of L-carnitine to additive solution-suspended red cells stored at 4°C reduces *in vitro* hemolysis and improves *in vivo* viability. *Transfusion*.

[15] Carl H., Chandni A., Neha K., Trishna S., Vani R. (2014). Curcumin as a modulator of oxidative stress during storage: a study on plasma. *Transfusion and Apheresis Science*.

[16] Shiva Shankar Reddy C. S., Subramanyam M. V. V., Vani R., Asha Devi S. (2007). *In vitro* models of oxidative stress in rat erythrocytes: effect of antioxidant supplements. *Toxicology in Vitro*.

[17] Møller P., Viscovich M., Lykkesfeldt J., Loft S., Jensen A., Poulsen H. E. (2004). Vitamin C supplementation decreases oxidative DNA damage in mononuclear blood cells of smokers. *European Journal of Nutrition*.

[18] Uzun F. G., Kalender Y. (2011). Protective effect of vitamins c and e on malathion-induced nephrotoxicity in male rats. *Gazi University Journal of Science*.

[19] Cross C. E., van der Vliet A., O'Neill C. A., Louie S., Halliwell B. (1994). Oxidants, antioxidants, and respiratory tract lining fluids. *Environmental Health Perspectives*.

[20] Halliwell B. (1996). Vitamin C: Antioxidant or pro-oxidant *in vivo*?. *Free Radical Research*.

[21] Moore G. L., Ledford M. E., Brummell M. R. (1981). Improved red blood cell storage using optional additive systems (OAS) containing adenine, glucose and ascorbate-2-phosphate. *Transfusion*.

[22] Moore G. L., Marks D. H., Carmen R. A. (1988). Ascorbate-2-phosphate in red cell preservation. Clinical trials and active components. *Transfusion*.

[23] Wood L. A., Beutler E. (1973). The effect of periodic mixing on the preservation of 2,3 diphosphoglycerate (2,3 DPG) levels in stored blood. *Blood*.

[24] Pallotta V., Gevi F., D'Alessandro A., Zolla L. (2014). Storing red blood cells with vitamin C and N-acetylcysteine prevents oxidative stress-related lesions: a metabolomics overview. *Blood Transfusion*.

[25] Stowell S. R., Smith N. H., Zimring J. C. (2013). Addition of ascorbic acid solution to stored murine red blood cells increases posttransfusion recovery and decreases microparticles and alloimmunization. *Transfusion*.

[26] Vani R., Reddy C. S. S. S., Asha Devi S. (2010). Oxidative stress in erythrocytes: a study on the effect of antioxidant mixtures during intermittent exposures to high altitude. *International Journal of Biometeorology*.

[27] Dodge J. T., Mitchell C., Hanahan D. J. (1963). The preparation and chemical characteristics of hemoglobin-free ghosts of human erythrocytes. *Archives of Biochemistry and Biophysics*.

[28] Misra H. P., Fridovich I. (1972). The role of superoxide anion in the autoxidation of epinephrine and a simple assay for superoxide dismutase. *The Journal of Biological Chemistry*.

[29] Aebi H. (1984). Catalase *in vitro*. *Methods in Enzymology*.

[30] Bar-Or D., Rael L. T., Lau E. P. (2001). An analog of the human albumin N-terminus (Asp-Ala-His-Lys) prevents formation of copper-induced reactive oxygen species. *Biochemical and Biophysical Research Communications*.

[31] Uchida K., Stadtman E. R. (1993). Covalent attachment of 4-hydroxynonenal to glyceraldehyde-3-phosphate dehydrogenase. A possible involvement of intra- and intermolecular cross- linking reaction. *The Journal of Biological Chemistry*.

[32] Witko V., Descamps-Latscha B. (1992). Microtiter plate assay for phagocyte-derived taurine-chloramines. *Journal of Clinical Laboratory Analysis*.

[33] Habeeb A. F. S. A. (1972). Reaction of protein sulfhydryl groups with Ellman's reagent. *Methods in Enzymology*.

[34] Lowry O. H., Rosenberg N. J., Farr A. L., Randall R. J. (1951). Protein measurement with the Folin phenol reagent. *The Journal of Biological Chemistry*.

[35] Soumya R., Vani R. (2015). CUPRAC–BCS and antioxidant activity assays as reliable markers of antioxidant capacity in erythrocytes. *Hematology*.

[36] Pandey K. B., Rizvi S. I. (2010). Markers of oxidative stress in erythrocytes and plasma during aging in humans. *Oxidative Medicine and Cellular Longevity*.

[37] Dreher D., Junod A. F. (1996). Role of oxygen free radicals in cancer development. *European Journal of Cancer*.

[38] Chen K., Suh J., Carr A. C., Morrow J. D., Zeind J., Frei B. (2000). Vitamin C suppresses oxidative lipid damage *in vivo*, even in the presence of iron overload. *The American Journal of Physiology—Endocrinology and Metabolism*.

[39] Mueller S., Riedel H.-D., Stremmel W. (1997). Direct evidence for catalase as the predominant H_2_O_2_-removing enzyme in human erythrocytes. *Blood*.

[40] Duarte T. L., Almeida G. M., Jones G. D. D. (2007). Investigation of the role of extracellular H_2_O_2_ and transition metal ions in the genotoxic action of ascorbic acid in cell culture models. *Toxicology Letters*.

[41] Padayatty S. J., Katz A., Wang Y. (2003). Vitamin C as an antioxidant: evaluation of its role in disease prevention. *Journal of the American College of Nutrition*.

[42] May J. M., Qu Z.-C., Cobb C. E. (2004). Human erythrocyte recycling of ascorbic acid: relative contributions from the ascorbate free radical and dehydroascorbic acid. *Journal of Biological Chemistry*.

[43] Oztas Y., Durukan I., Unal S., Ozgunes N. (2012). Plasma protein oxidation is correlated positively with plasma iron levels and negatively with hemolysate zinc levels in sickle-cell anemia patients. *International Journal of Laboratory Hematology*.

[44] Berlett B. S., Stadtman E. R. (1997). Protein oxidation in aging, disease, and oxidative stress. *The Journal of Biological Chemistry*.

[45] Witko-Sarsat V., Friedlander M., Khoa T. N. (1998). Advanced oxidation protein products as novel mediators of inflammation and monocyte activation in chronic renal failure. *Journal of Immunology*.

[46] Rizvi S. I., Pandey K. B., Jha R., Maurya P. K. (2009). Ascorbate recycling by erythrocytes during aging in humans. *Rejuvenation Research*.

[47] Herrero E., Ros J., Bellí G., Cabiscol E. (2008). Redox control and oxidative stress in yeast cells. *Biochimica et Biophysica Acta—General Subjects*.

[48] Türkes S., Korkmaz Ö., Korkmaz M. (2003). Time course of the age-related alterations in stored blood. *Biophysical Chemistry*.

